# Assessment of Temperature-Independent Resistance against Bacterial Wilt Using Major QTL in Cultivated Tomato (*Solanum lycopersicum* L.)

**DOI:** 10.3390/plants11172223

**Published:** 2022-08-27

**Authors:** Jeyun Yeon, Ngoc Thi Le, Sung-Chur Sim

**Affiliations:** 1 Department of Bioresources Engineering, Sejong University, Seoul 05006, Korea; 2 Plant Engineering Research Institute, Sejong University, Seoul 05006, Korea

**Keywords:** vegetable, bacterial disease, host resistance, QTL, breeding

## Abstract

Bacterial wilt (*Ralstonia solanacearum*) is a devastating disease of cultivated tomato resulting in severe yield loss. Since chemicals are often ineffective in controlling this soil-borne pathogen, quantitative trait loci (QTL) conferring host resistance have been extensively explored. In this study, we investigated effects of ambient temperature and major QTL on bacterial wilt resistance in a collection of 50 tomato varieties. The five-week-old seedlings were inoculated using the race 1 (biovar 4 and phylotype I) strain of *R. solanacearum* and placed at growth chambers with three different temperatures (24 °C, 28 °C, and 36 °C). Disease severity was evaluated for seven days after inoculation using the 1–5 rating scales. Consistent bacterial wilt resistance was observed in 25 tomato varieties (R group) with the means of 1.16–1.44 for disease severity at all three temperatures. Similarly, 10 susceptible varieties with the means of 4.37–4.73 (S group) were temperature-independent. However, the other 15 varieties (R/S group) showed moderate levels of resistance at both 24 °C (1.84) and 28 °C (2.16), while they were highly susceptible with a mean of 4.20 at 36 °C. The temperature-dependent responses in the R/S group were supported by pairwise estimates of the Pearson correlation coefficients. Genotyping for three major QTL (*Bwr-4*, *Bwr-6* and *Bwr-12*) found that 92% of varieties in the R group had ≥ two QTL and 40% of varieties in the R/S group had one or two QTL. This suggests that these QTL are important for stability of resistance against bacterial wilt at high ambient temperature. The resulting 25 varieties with temperature-independent resistance will be a useful resource to develop elite cultivars in tomato breeding programs.

## 1. Introduction

Bacterial wilt, caused by *Ralstonia solanacearum*, is one of the most important diseases with a wide host range including major crop species, such as tomato, potato, eggplant, pepper, and tobacco in the Solanaceae family [1]. This soil-borne pathogen has a worldwide distribution with genetic diversity and is classified into five races and six biovars based on host range and biochemical properties [1,2]. Four phylotypes of *R. solanacearum* were also proposed according to geographical origins [3]. For cultivated tomato, race 1 (phylotype I and II) and race 3 (phylotype II) are the most virulent in temperate regions [4]. Infection of bacterial wilt via physical wounds or natural openings of roots leads to lethal wilting symptoms in host plants due to rapid colonization in the vascular system, especially xylem vessels [5,6].

Chemical control is often ineffective to eliminate the soil-borne pathogen in infested tomato fields due to bacterial localization in deep soils [2]. In addition, use of chemicals for soil disinfection can be harmful to the environment. An alternative control strategy is to use host resistance that provides an environment-friendly and cost-effective method [7]. However, environmental factors, especially temperature, is known to affect resistance against plant diseases [1,8,9]. For bacterial wilts, high temperatures of 30–35 °C were related with an increase of susceptibility in several hosts [1,10,11,12,13]. In tomato, the variety VC 48 was consistently resistant to three soil temperatures (26, 30, and 32 °C), while the other three varieties VC 8, VC 9, and VC 11 showed different responses depending on temperatures: resistant at 26 °C and susceptible at 32 °C [12]. Similarly, several resistant varieties were severely infected by bacterial wilt at the ambient temperature of 35 °C [11,13]. Therefore, genetic dissection of temperature-dependent resistance is essential to improve bacterial wilt resistance in tomato breeding programs.

Bacterial wilt resistance is known to be polygenic and there are varieties with different levels of resistance in cultivated tomato [1,14,15]. For example, the ‘Hawaii7996 (Ha7996)’ and ‘Hawaii7998 (Ha7998)’ varieties are important sources of polygenic resistance [10,15,16,17]. These varieties were often used to investigate quantitative trait loci (QTL) associated with bacterial wilt resistance [4,14,18,19,20,21]. Of the QTL identified in the previous studies, two major QTL, named *Bwr-6* and *Bwr-12*, were mapped on chromosomes 6 and 12, explaining 11.5–22.2% and 17.9–56.1% of the phenotypic variance, respectively [20]. Recently, a genome-wide association study (GWAS) in a collection of diverse tomato varieties identified an additional major QTL on chromosome 4 along with *Bwr-6* and *Bwr-12* [22]. These QTL may be associated with stable resistance against bacterial wilt at high temperatures.

Breeders have made large efforts to improve bacterial wilt resistance in elite tomato cultivars. However, breakdown of resistance by high temperature needs to be comprehensively resolved for this breeding goal. The present study was conducted to (1) evaluate temperature-dependent resistance against the race 1 (biovar 4 and phylotype I) strain of *R. solanacearum* in a collection of 50 tomato varieties at three ambient temperatures and (2) investigate effects of three major QTL (*Bwr-4*, *Bwr-6*, and *Bwr-12*) on stable resistance using the molecular markers associated with these loci. The 50 varieties were divided into three groups (R, R/S, and S) based on levels of bacterial wilt resistance at different temperatures. Of these, the R/S group (*n* = 15) showed temperature-dependent resistance and nine varieties of this group were with none of the major QTL. The results from this study will be useful for genetic dissection of bacterial wilt resistance and developing an efficient control strategy for this soil-borne disease in tomato.

## 2. Results

### 2.1. Effect of Temperature for Bacterial Wilt Resistance

**Figure 1:**
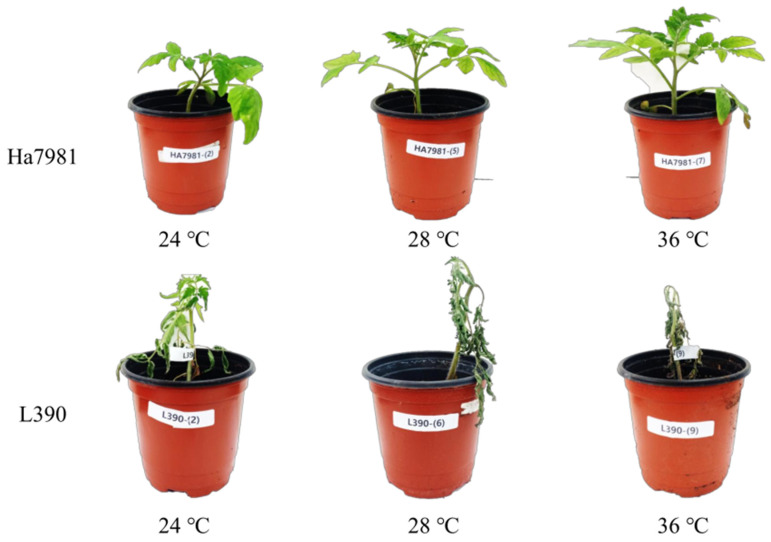
Disease severity of the resistance control ‘Ha7981’ and susceptible control ‘L390’ on day 7 after inoculation using the WR-1 strain (race 1, biovar 4, and phylotype I) of bacterial wilt at three different temperatures (24 °C, 28 °C, and 36 °C).

**Figure 2:**
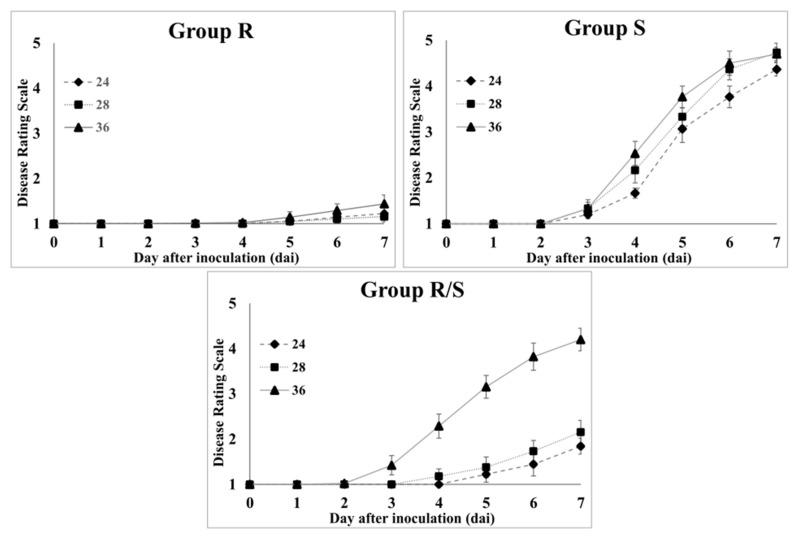
Bacterial wilt progress in the three groups (R, R/S, and S) of 50 tomato varieties at three ambient temperatures (24 °C, 28 °C, and 36 °C). Each group consisted of 25 (R group), 15 (R/S group), and 10 (S group) varieties. The five-week-old seedlings were inoculated with the WR-1 strain (race 1, biovar 4, and phylotype I) and disease progress was scored for seven days after inoculation using the 1–5 rating scale (1 = no wilting symptom and 5 = plant died).

The seedlings of 50 tomato varieties were evaluated along with two controls for bacterial wilt resistance against the WR-1 strain (race 1, biovar 4, and phylotype I) at growth chambers with different ambient temperatures (24 °C, 28 °C, and 36 °C). The resistant and susceptible controls, ‘Ha7981’ and ‘L390’ showed consistent responses against the WR-1 strain with no significant difference for disease severity between temperatures, respectively (Table 1 and Figure 1). The 50 tomato varieties were divided into three groups (R, R/S, and S) based on their responses to the bacterial wilt strain at three different temperatures. The R group consisting of 25 varieties showed constantly high levels of resistance at all temperatures for seven days after inoculation (Table 1 and Figure 2). Their means of disease severity on day 7 after inoculation were 1.23 at 24 °C, 1.16 at 28 °C, and 1.44 at 36 °C, which were not significantly different at *p* < 0.05. Furthermore, there was no significant difference for levels of resistance relative to the resistant control. The 10 varieties of S group showed highly susceptible responses with the means of 4.37 at 24 °C, 4.73 at 28 °C, and 4.70 at 36 °C for disease severity, which were not significantly different from 5.00 of the L390 variety (susceptible control) at *p* < 0.05 (Table 1 and Figure 2). In the third group (R/S), we found that resistance levels against bacterial wilt were temperature-dependent. The 15 varieties in this group showed moderate levels of resistance with mean disease severities of 1.84 at 24 °C and 2.16 at 28 °C, which were significantly different from the temperature-independent groups, R and S at *p* < 0.05 (Table 1 and Figure 2). However, susceptible responses were observed at 36 °C in this group with a mean disease severity of 4.20 on day 7 after inoculation, which was not significantly different from the mean disease severities of 4.37 (S group) and 5.00 (the susceptible control). 

In addition, the Pearson correlation coefficients were calculated for disease severity between temperatures in the 50 tomato varieties (Table 2). A high level of correlation with the coefficient of 0.952 was found between 24 °C and 28 °C, while the correlation coefficients between low and high temperatures were 0.713 (24 °C vs. 36 °C) and 0.765 (28 °C vs. 36 °C). As observed with the means of disease severity, the pairwise estimates of coefficients also indicated presence of temperature-dependent resistance in the tomato varieties. 

### 2.2. Genotyping of Tomato Varieties for Major QTL

The 50 tomato varieties and two controls were genotyped using the SNP markers that were previously developed for three major QTL associated with bacterial wilt resistance including *Bwr-4*, *Bwr-6*, and *Bwr-12* (Table 3). All of these QTL were present in the resistant control ‘Ha7981’, while none of these were in the susceptible control ‘L390’. As the resistant control, 25 varieties in the R group showed high levels of resistance regardless of temperature. Of these, we found 23 varieties (92%) with ≥ two major QTL: 17 varieties with *Bwr-4* and *Bwr-12*, two with *Bwr-4* and *Bwr-6*, and four with all three QTL (Table 3 and Table 4). There were two additional varieties without any of the major QTL in the R group that still showed temperature-independent resistance. In the S group, there was no variety with any of the major QTL (Table 3 and Table 4). Although the R/S group showed temperature-dependent resistance, only six of 15 varieties (40%) were determined with one or two QTL: three varieties with *Bwr-4*, one with *Bwr-6*, one with *Bwr-4* and *Bwr-6*, and one with *Bwr-4* and *Bwr-12* (Table 3 and Table 4). For the other nine varieties, no major QTL were found, but moderate levels of resistance were still shown at 24 °C and 28 °C. Their means of disease severity ranged from 1.33 to 2.67 that were similar to the six varieties with one or two QTL in the R/S group (Table 3).

## 3. Discussion

Bacterial wilt is a major limiting factor for tomato production worldwide because this disease rapidly colonizes the xylem after infection and leads to severe wilting symptoms in plants. Disease incidence and severity are affected by environmental factors, especially temperature [1]. High temperature has been known to be associated with an increase in disease severity and host resistance is often unstable in this environmental condition [11,12,13]. Several tomato varieties, which were grown at different soil temperatures, showed resistance at 26 °C but became susceptible at 32 °C [12]. In addition, severe wilting symptoms were developed in the known resistant tomato varieties including Ha7996 and Ha7998 at 35 °C for light cycle and 28 °C for dark cycle after inoculation using race 1 strains [11]. Similarly, Singh et al. [13] indicated that tomato cultivars showed 97–99% disease intensity at 35 °C on day 11 after inoculation, while no symptom was observed in both cultivars at 20 °C until day 60. In the present study, we validated the effect of temperature on bacterial wilt resistance in a collection of 50 tomato varieties using the highly virulent WR-1 strain (race 1, biovar4, and phylotype I) that leads to rapid disease progress. Instability of resistance against this strain was demonstrated in 15 of 50 tomato varieties at 36 °C for light cycle and 24 °C for dark cycle. These varieties (R/S group) were moderately resistant at relatively low temperature conditions (24 °C and 28 °C for light cycle). Interestingly, 25 tomato varieties (R group) showed high levels of resistance at both low and high temperatures, while the other 10 varieties (S group) resulted in lethal wilting on day 7 after inoculation. This result suggests that the 25 varieties will be useful for improving bacterial wilt resistance in tomato breeding programs. 

As an effective control strategy, host resistance against bacterial wilt has been investigated in tomato. Genetic mapping studies identified a number of QTL associated with resistance on different chromosomes [4,14,18,19,20,21,23]. Of these, *Bwr-4*, *Bwr-6*, and *Bwr-12* were reported as major QTL and molecular markers were developed for selection of these QTL in our previous study [22]. These SNP markers were used to genotype the three groups of 50 tomato varieties and inspect roles of the major QTL for stable resistance regardless of temperature. This analysis indicated that these QTL were found with higher frequency in the R group relative to the R/S and S groups. In the R group, 92% of tomato varieties were with ≥ two major QTL, including 17 varieties with both *Bwr-4* and *Bwr-12*. In contrast, 40% of varieties in the R/S group possessed one or two QTL, while the other varieties had none of the major QTL, suggesting that these QTL, especially *Bwr-4* and *Bwr-12*, are important for temperature-independent resistance against bacterial wilt. We found that two varieties with none of these QTL in the R group showed still high levels of resistance at high temperature. In these varieties, the SNP markers may be identical by state but not identical by descent because of more opportunities for recombination between marker and QTL. In addition, two varieties in the R/S group were with two QTLs: one with *Bwr-4* and *Bwr-6*, and another with *Bwr-4* and *Bwr-12*. It is possible that temperature-independent resistance against bacterial wilt is derived from a large number of loci along with the three major QTL. Therefore, their instability of resistance at high temperature may be explained with lack of additional major or minor QTL. Although many efforts have been made to detect QTL for bacterial wilt resistance in previous studies, there may be still uncovered QTL with major or minor effects. Identification of novel QTL is required to expand our understanding for bacterial wilt resistance. 

In conclusion, our study demonstrated that bacterial wilt resistance against the WR-1 strain (race 1, biovar 4, and phylotype I) was affected by high ambient temperature, resulting in breakdown of resistance at 36 °C in cultivated tomato. However, 25 of 50 tomato varieties used in this study showed high levels of resistance at both low and high temperatures. Genotyping for three major QTL, which were identified on chromosomes 4 (*Bwr-4*), 6 (*Bwr-6*), and 12 (*Bwr-12*), revealed that ≥ two major QTL were present in the majority of varieties with temperature-independent resistance. None of these QTL was found in the 10 susceptible varieties regardless of temperatures. In addition, we found one or two QTL in 40% of varieties with temperature-dependent resistance. This result suggests that the major QTL are responsible for temperature-independent resistance against bacterial wilt. Since additional QTL may be required for stability of resistance at high temperature, the conclusion needs to be treated with caution. The resistant tomato varieties from this study, which are inbred lines, can be utilized for further genetic dissection of bacterial wilt resistance and development of elite cultivars with durable resistance in tomato.

## 4. Materials and Methods

### 4.1. Plant Materials

A total of 50 tomato varieties were collected from a private breeding program. These inbred breeding lines represented different levels of resistance against bacterial wilt including resistant and susceptible lines. In addition, the varieties ‘Ha7981’ and ‘L390’ were used as resistant and susceptible controls along with the 50 inbred lines for disease assay. Seedlings of these varieties were grown in a growth chamber at 28 °C for five weeks after sowing to assess effect of temperature on host resistance against bacterial wilt. Disease assay with five-week-old seedlings was conducted with three replications for each of the 50 inbred lines plus resistant and susceptible controls. The replicates were randomized in a temperature treatment.

### 4.2. Seedling Assay for Evaluation of Bacterial Wilt Resistance

A virulent strain of *R. solanacearum*, WR-1 (race 1, biovar 4, and phylotype I), was isolated from an infected tomato in the Republic of Korea by the National Institute of Horticultural & Herbal Science (NIHHS). For inoculum preparation, the WR-1 strain was cultured on the Difco^TM^ nutrient broth medium that contained 5 g/L peptone and 3 g/L beef extract (BD, Sparks, MD, USA). After incubation at 28 °C for 48 h, bacterial cells were collected and resuspended in sterile, double-distilled waters. Concentration of bacterial cells in the suspension was then standardized to OD_600_ = 0.3 (10^8^ CFU/mL) using the NanoDrop™ One spectrophotometer (ThermoFisher Scientific, Waltham, MA, USA). The roots of five-week-old seedlings were first wounded by cutting, then dipped in the bacterial suspension for 30 min for artificial inoculation [4]. Three seedlings per tomato variety were inoculated and transplanted into individual plastic pots (Ø10 × 10 cm) filled with fresh potting soils. The inoculated plants were incubated in three different growth chambers at 24 °C, 28 °C, and 36 °C with 14 h for light cycle and 24 °C with 10 h for dark cycle per day. Disease severity was monitored daily for seven days after inoculation (dai) using the 1 to 5 rating scales, where 1 = no wilting symptom, 2 = one or two leaves wilted, 3 = more than three wilted leaves, 4 = all leaves wilted, and 5 = plant died [24]. With the resulting disease scores of three replicates per variety, analysis of variance (ANOVA) was conducted using the general linear model and mean separation of disease severity was evaluated using the least significant difference (LSD) test in R program (https://www.r-project.org/ (accessed on 1 February 2022)). 

### 4.3. High Resolution Melting Analysis for Genotyping

Genomic DNA of each tomato variety was isolated from fresh, young leaves of seedlings using a modified cetyl trimethyl ammonium bromide (CTAB) method [25]. DNA concentration was adjusted to 50 ng/μL using the NanoDrop™ One spectrophotometer. The SNP markers for identification of three major QTL (*Bwr-4*, *Bwr-6*, and *Bwr-12*) were developed for high resolution melting (HRM) analysis in our previous study [22]. Therefore, these markers were used to determine presence of major QTL in the 50 tomato varieties and HRM analysis was conducted using the LightCyler^®^ 96 real-time PCR System (Roche Diagnostics, Indianapolis, IN, USA). A total 10 µL of reaction mixture was prepared with 30–50 ng of DNA template, 10 µM of each primer, 25 mM MgCl2, and 5 µL of 2X LightCycler^®^ 480 HRM master mix including a SybrGreen fluorescent dye. PCR amplification with this reaction mixture was first conducted with initial denaturation for 10 min at 95 °C, 80 cycles of 10 s at 95 °C, 20 s at 54 °C, 10 s at 72 °C. Sequentially, the resulting PCR amplicons were denatured for 1 min at 95 °C and then held for 1 min at 40 °C to anneal the DNA duplexes. These steps were followed by a melting curve ranging from 65 to 95 °C with temperature increments of 4.4 °C per second. Melting curves for SNP calling were generated from the fluorescence data using the LightCycler^®^96 Application Software v1.1 (Roche Diagnostics).

## Figures and Tables

**Table 1:** The seedling assay of bacterial wilt disease at three different ambient temperatures in 50 tomato varieties and controls.

Group ^a^	No. of Variety	Temperature (°C)	Mean of Disease Severity ± SD ^b^	LSD Grouping ^c^
R	25	24	1.23 ± 0.23	a
		28	1.16 ± 0.24	a
		36	1.44 ± 0.39	a
R/S	15	24	1.84 ± 0.35	b
		28	2.16 ± 0.52	b
		36	4.20 ± 0.50	c
S	10	24	4.37 ± 0.29	cd
		28	4.73 ± 0.41	d
		36	4.70 ± 0.29	d
Resistant control	1	24	1.00 ± 0.00	a
		28	1.00 ± 0.00	a
		36	1.00 ± 0.00	a
Susceptible control	1	24	5.00 ± 0.00	d
		28	5.00 ± 0.00	d
		36	5.00 ± 0.00	d

^a^ R and S = temperature-independent resistant (R) and susceptible (S) varieties, R/S = temperature-dependent resistant varieties, resistant control = Ha7981, and susceptible control = L390. ^b^ Disease severity was scored on day 7 after inoculation of the WR-1 strain (race 1, biovar 4, and phylotype I) using the 1–5 scales (1 = no wilting symptom and 5 = plant died). Disease scores of three seedlings per tomato variety were used for analysis of variance (ANOVA) and mean separation between groups. SD = standard deviation. ^c^ Mean comparisons were conducted using the least significant difference (LSD) test. Means with the same letter are not significantly different at *p* < 0.05.

**Table 2:** Correlation of bacterial wilt severity between temperatures in 50 tomato varieties.

Temperature	Pearson Correlation Coefficient	
	28 °C	36 °C
24 °C	0.952	0.713
28 °C	-	0.765

**Table 3:** Disease severity and genotypes of 50 tomato varieties for three major QTL associated with bacterial wilt resistance.

Variety	Group	Mean of Disease Severity ± SD ^a^			Major QTL Genotype ^b^		
		24 °C	28 °C	36 °C	*Bwr-4*	*Bwr-6*	*Bwr-12*
BP1151	R	1.00 ± 0.00	1.00 ± 0.00	1.33 ± 0.47	R	R	R
BP1165	R	1.33 ± 0.47	1.33 ± 0.47	1.00 ± 0.00	R	R	S
BP1189	R	1.33 ± 0.47	1.00 ± 0.00	1.00 ± 0.00	R	R	R
BP1198	R	1.33 ± 0.47	1.00 ± 0.00	1.33 ± 0.47	R	R	S
BP1206	R	1.33 ± 0.47	1.33 ± 0.47	1.33 ± 0.47	R	S	R
BP1207	R	1.00 ± 0.00	1.00 ± 0.00	1.33 ± 0.47	R	S	R
BP1208	R	1.33 ± 0.47	1.33 ± 0.47	1.67 ± 0.94	R	S	R
BP1209	R	1.00 ± 0.00	1.00 ± 0.00	2.00 ± 0.82	S	S	S
BP1215	R	1.67 ± 0.47	1.33 ± 0.47	1.33 ± 0.47	R	S	R
BP1216	R	1.00 ± 0.00	1.00 ± 0.00	1.00 ± 0.00	R	S	R
BP1223	R	1.00 ± 0.00	1.33 ± 0.47	1.33 ± 0.47	R	S	R
BP1227	R	1.00 ± 0.00	1.00 ± 0.00	1.67 ± 0.47	R	S	R
BP1233	R	1.33 ± 0.47	1.00 ± 0.00	2.00 ± 0.82	R	S	R
BP1234	R	1.00 ± 0.00	1.00 ± 0.00	1.67 ± 0.94	R	S	R
BP1246	R	1.00 ± 0.00	1.00 ± 0.00	1.33 ± 0.47	R	S	R
BP1249	R	1.33 ± 0.47	1.00 ± 0.00	1.33 ± 0.47	R	S	R
BP1250	R	1.33 ± 0.47	1.67 ± 0.47	1.33 ± 0.47	R	S	R
BP1258	R	1.33 ± 0.47	1.67 ± 0.94	1.67 ± 0.94	S	S	S
BP1261	R	1.67 ± 0.94	1.00 ± 0.00	1.00 ± 0.00	R	S	R
BP1278	R	1.33 ± 0.47	1.00 ± 0.00	2.33 ± 0.94	R	S	R
BP1296	R	1.00 ± 0.00	1.67 ± 0.47	2.33 ± 1.25	R	S	R
BP1299	R	1.00 ± 0.00	1.00 ± 0.00	1.33 ± 0.47	R	R	R
BP1300	R	1.33 ± 0.47	1.33 ± 0.47	1.00 ± 0.00	R	R	R
BP1342	R	1.67 ± 0.94	1.00 ± 0.00	1.33 ± 0.47	R	S	R
BP1344	R	1.00 ± 0.00	1.00 ± 0.00	1.00 ± 0.00	R	S	R
BP1163	R/S	1.33 ± 0.47	2.00 ± 0.82	4.33 ± 0.94	S	S	S
BP1187	R/S	1.67 ± 0.47	2.33 ± 0.47	3.67 ± 0.47	S	S	S
BP1195	R/S	1.67 ± 0.47	1.67 ± 0.94	4.00 ± 0.00	R	S	S
BP1200	R/S	2.33 ± 0.94	1.67 ± 0.47	4.00 ± 0.82	R	S	S
BP1201	R/S	2.00 ± 1.41	3.00 ± 0.82	4.67 ± 0.47	R	R	S
BP1202	R/S	2.33 ± 0.47	2.33 ± 0.47	3.67 ± 0.94	S	S	S
BP1210	R/S	1.67 ± 0.94	2.67 ± 0.47	3.67 ± 0.47	S	S	S
BP1219	R/S	2.00 ± 0.00	3.00 ± 0.82	4.00 ± 0.00	S	R	S
BP1225	R/S	1.67 ± 0.47	2.33 ± 0.47	5.00 ± 0.00	R	S	S
BP1251	R/S	2.00 ± 0.82	1.67 ± 0.94	4.33 ± 0.94	S	S	S
BP1280	R/S	2.00 ± 0.00	2.00 ± 0.82	4.67 ± 0.47	R	S	R
BP1287	R/S	1.33 ± 0.47	2.00 ± 0.82	4.67 ± 0.47	S	S	S
BP1316	R/S	2.00 ± 0.82	2.67 ± 1.70	3.67 ± 0.47	S	S	S
BP1340	R/S	1.33 ± 0.47	1.33 ± 0.47	3.67 ± 0.47	S	S	S
BP1341	R/S	2.33 ± 0.47	1.67 ± 0.47	5.00 ± 0.00	S	S	S
BP1161	S	4.67 ± 0.47	5.00 ± 0.00	4.33 ± 0.94	S	S	S
BP1217	S	4.33 ± 0.47	4.67 ± 0.47	5.00 ± 0.00	S	S	S
BP1241	S	4.00 ± 0.82	4.00 ± 0.00	4.67 ± 0.47	S	S	S
BP1267	S	4.33 ± 0.47	5.00 ± 0.00	4.33 ± 0.94	S	S	S
BP1268	S	4.33 ± 0.47	5.00 ± 0.00	5.00 ± 0.00	S	S	S
BP1269	S	4.33 ± 0.47	4.67 ± 0.47	5.00 ± 0.00	S	S	S
BP1312	S	4.33 ± 0.47	5.00 ± 0.00	4.33 ± 0.47	S	S	S
BP1321	S	5.00 ± 0.00	5.00 ± 0.00	4.67 ± 0.47	S	S	S
BP1334	S	4.00 ± 0.82	4.00 ± 0.82	4.67 ± 0.47	S	S	S
BP1339	S	4.33 ± 0.47	5.00 ± 0.00	5.00 ± 0.00	S	S	S
Ha7981	Resistant control	1.00 ± 0.00	1.00 ± 0.00	1.00 ± 0.00	R	R	R
L390	Susceptible control	5.00 ± 0.00	5.00 ± 0.00	5.00 ± 0.00	S	S	S

^a^ Disease severity was scored on day 7 after inoculation of the WR-1 strain (race 1, biovar 4, and phylotype I) using the 1–5 scales (1 = no wilting symptom and 5 = plant died). SD = standard deviation. ^b^ Major QTL genotypes in the 50 varieties and controls were determined using the SNP markers developed in the study of Nguyen et al. [22]. R and S indicate resistant and susceptible alleles for major QTL, respectively.

**Table 4:** Summary of genotyping for three major QTL associated with bacterial wilt resistance in 50 tomato varieties.

QTL	Number of Variety ^a^		
	R	R/S	S
*Bwr-4*	0	3	0
*Bwr-6*	0	1	0
*Bwr-12*	0	0	0
*Bwr-4* & *6*	2	1	0
*Bwr-4* & *12*	17	1	0
*Bwr-6* & *12*	0	0	0
*Bwr-4, 6,* & *12*	4	0	0
None	2	9	10
Total	25	15	10

^a^ Number of variety in three groups (R, R/S, and S) was determined based on presence of three major QTL. The varieties with only one of these QTL are separated from those with multiple QTL (two or three QTL). None indicates the varieties without any of these QTL.

## Data Availability

Not applicable.
